# ChIAPoP: a new tool for ChIA-PET data analysis

**DOI:** 10.1093/nar/gkz062

**Published:** 2019-02-08

**Authors:** Weichun Huang, Mario Medvedovic, Jingwen Zhang, Liang Niu

**Affiliations:** 1 National Exposure Research Laboratory, Environmental Protection Agency, Research Triangle Park, NC 27709, USA; 2 Division of Biostatistics and Bioinformatics, Department of Environmental Health, College of Medicine, University of Cincinnati, Cincinnati, OH 45267, USA; 3 National Key Laboratory of Crop Genetic Improvement, Hubei Key Laboratory of Agricultural Bioinformatics, College of Informatics, Huazhong Agricultural University, Wuhan 430070, China

## Abstract

Chromatin Interaction Analysis by Paired-End Tag Sequencing (ChIA-PET) is a popular assay method for studying genome-wide chromatin interactions mediated by a protein of interest. The main goal of ChIA-PET data analysis is to detect interactions between DNA regions. Here, we propose a new method and the associated data analysis pipeline, ChIAPoP, to detect chromatin interactions from ChIA-PET data. We compared ChIAPoP with other popular methods, including a hypergeometric model (used in ChIA-PET tool), MICC (used in ChIA-PET2), ChiaSig and mango. The results showed that ChIA-PoP performed better than or at least as well as these top existing methods in detecting true chromatin interactions. ChIAPoP is freely available to the public at https://github.com/wh90999/ChIAPoP.

## INTRODUCTION

Chromatin Interaction Analysis by Paired-End Tag Sequencing (1) (ChIA-PET), first introduced in 2009, is an experimental assay for studying genome-wide chromatin interactions mediated by a protein of interest. It has been widely used to study different proteins in different genomes, such as oestrogen receptor alpha in the human genome (2), RNA polymerase II in the human genome (3), CCCTC-binding factor (CTCF) in the mouse genome (4), etc. Recently, an improved (long read) ChIA-PET protocol was introduced (5) and has since been used in a study of genome-wide chromatin interactions mediated by CTCF (6) in the human genome.

A typical ChIA-PET experiment generates tens of millions of paired reads. Each read contains a tag (a piece of DNA sequence from the related genome) and a linker sequence (barcode). The tags generated by the original protocol are short (usually $20 \pm 1$ base pairs), while the tags generated by the improved protocol are typically longer (the lengths vary and are up to 150 or 250 base pairs). By mapping the paired tags to the reference genome, potentially interactive pairs of DNA regions, together with the counts of paired tags mapped to the pairs, can be identified. For the sake of simplicity, here we call such DNA regions and potentially interactive pairs as anchor regions and potential pairs, respectively. Among these potential pairs, some are true interactive ones containing an interaction signal, while the others are of no interactions and are random noise. Thus, the main goal of ChIA-PET data analysis is to distinguish signal from noise using observed count data for potential pairs.

To distinguish signal from noise, many tools have been proposed (7–11). Among them, the ChIA-PET tool (7), ChiaSig (8), mango (9) and ChIA-PET2 (12) are popular ones. The ChIA-PET tool, which is the first tool for ChIA-PET data analysis, uses a hypergeometric (HG) distribution to model count data. The HG model accounts for the sequencing depth, also called sequencing bias, of individual anchor regions. The underlying assumption is that the random (i.e. no true interaction) pairing chance of two anchor regions increases as the sequencing coverage depth of the two anchor regions increases. ChiaSig improves the ChIA-PET tool by using a more general non-central HG distribution to model count data. It takes an additional factor, the genomic distance between two paired anchor regions within a chromosome, into consideration. The underlying assumption is that the random pairing chance of two anchor regions decreases as the genomic distance between the two anchor regions increases. Mango is similar to ChiaSig, but uses a binomial model instead of a non-central HG model. A limitation of Mango is that it does not model count data for potential pairs of anchor regions from two different chromosomes. While both ChiaSig and mango markedly reduce false positive hits, they also potentially eliminate many true interactions when compared to the ChIA-PET tool (9). ChIA-PET2 uses MICC (10), an R package based on a Bayesian mixture model of count data, to identify chromatin interactions. For the same input data, MICC reports a slightly different set of significant pairs at each run, as its algorithm employs random number generators.

Here, we present a new method and the associated pipeline tool, Chromatin Interaction Analysis with Positive Poisson (ChIAPoP), to distinguish signal from noise in ChIA-PET data of the original protocol. It is an integrated pipeline that requires only two input sequencing read data files to start analysis. ChIAPoP takes into consideration the sequencing bias of anchor regions and the genomic distances between two paired anchor regions. Tested on two publicly available ChIA-PET datasets, the K562 RNA polymerase II and MCF7 RNA polymerase II data in (3), we showed that ChIAPoP fitted count data well and that it performed better than or at least as well as the top existing methods including HG (ChIA-PET tool), ChiaSig, mango and MICC (ChIA-PET2). ChIAPoP was implemented in R and is freely available as a fully documented R package at GitHub. The R package depends on bowtie (13) (for read alignment), MACS2 (14) (for peak calling) and a few (mostly Bioconductor) R packages, e.g. ShortRead (15), GenomicAlignments (16) and GenomeInfoDb (17).

## MATERIALS AND METHODS

### Overview

ChIAPoP takes two read files (paired, in the FASTQ format) from the original ChIA-PET protocol (1) as the input and outputs the chromosome locations, count, *P*-value and False Discovery Rate (FDR)-adjusted *P*-value for each potentially interactive anchor region pair. The pipeline consists of six steps. In step 1, the linkers are removed from the raw reads and the resulting paired reads are separated into two categories: regular read pairs (with both linkers of the same type) and chimeric read pairs (with two linkers of different types). This step generates four read files, including two read files for regular read pairs and two read files for chimeric read pairs. In step 2, the four read files are aligned to a reference genome using bowtie one by one. This step generates four alignment files (in the SAM format). In step 3, the regular alignment files are processed to filter out alignment pairs with at least one unaligned read and duplicated alignment pairs. In addition, the strand orientation of each alignment pair is reversed to make it suitable for peak calling in the next step. The two chimeric alignment files are processed in the same way. This step generates four processed alignment files. In step 4, the four processed alignment files are used for peak calling using MACS2. The four files are treated as independent single-end alignment files and the pairing information is ignored. This step generates a file (in the BED format) of read peaks. In step 5, the anchor regions are built using the detected peaks and the single-end (processed) alignments are extended up to a typical fragment length. Then, the number of regular fragment pairs that connect (i.e. overlap) any two different anchor regions are counted and a regular count table (for all anchor region pairs with non-zero counts) is generated. Similarly, the number of chimeric fragment pairs that connect any two anchor regions (the two anchor regions can be identical) are counted and a chimeric count table (for all anchor region pairs with non-zero counts) is generated. In step 6, pairs of anchor regions in the regular count table, i.e. potential pairs, are divided into two groups: inter-chromosomal pairs and intra-chromosomal pairs. In each group, each pair is assigned a *P*-value using a positive Poisson (i.e. zero truncated Poisson) distribution with a pair-specific parameter ($\lambda$). Benjamini–Hochberg procedure (18) is then applied to the two groups (as a whole) to calculate the FDR adjusted *P*-values. Please see Supplementary Figure S1 in the Supplementary Data for the flow chart of ChIAPoP pipeline.

### Positive Poisson model

For a given potential pair, we assume that the observed count, under the null hypothesis that there is no interaction between the two anchor regions, follows a pair-specific positive Poisson distribution. Because of that, under the null hypothesis, random pairing of two anchor regions from different chromosomes is affected only by the sequencing bias of the two regions, while that of two anchor regions from the same chromosome is affected by both their sequencing bias and the genomic distance, we model inter-chromosomal and intra-chromosomal count data separately. Here, the sequencing bias of an anchor region pair (either a potential pair, or a chimeric pair with two different anchor regions) is defined as the product of sequencing bias of the two anchor regions, where the sequencing bias of an anchor region is the number of fragments that overlap with the anchor region (excluding the fragments from those regular fragment pairs with both fragments overlap with the anchor region). If a pair consists of two identical anchors (only possible in a chimeric pair), then the sequencing bias of the pair is defined as a half of the square of sequencing bias of the anchor region. It can be shown that, if observed counts follow a HG model (which is the null model of the ChIA-PET tool), the expected count of a pair of anchor regions is proportional to the sequencing bias of the pair. For convenience in the following discussion, we use ${n_c}$, ${n_{{\rm inter}}}$ and ${n_{{\rm intra}}}$ to represent the number of chimeric pairs, the number of inter-chromosomal pairs and the number of intra-chromosomal pairs, respectively.

To estimate the positive Poisson parameter ${\lambda _i}$ for the inter-chromosomal pair $i$ ($1 \le i \le {n_{{\rm inter}}}$), we first fit a positive Poisson regression

\begin{equation*}\ \log \left( {{\lambda _j}} \right) = {\beta _0}\ + {\beta _1} \cdot {\rm{log}}\left( {{\rm seq.bia}{{\rm s}_j}} \right)\end{equation*}

to the chimeric data, where $j\ = \ 1,\ 2,\ \cdots ,\ {n_c}$ is the index for the chimeric pairs, ${\lambda _j}$ is the positive Poisson parameter for chimeric pair $j$ and ${\rm seq.bia}{{\rm s}_j}$ is the sequencing bias for chimeric pair $j.$ Then we use the estimated parameters (${\hat{\beta }_0}$ and ${\hat{\beta }_1}$) to estimate ${\lambda _i},$ i.e. ${\hat{\lambda }_i} = {e^{{{\hat{\beta }}_0} + {{\hat{\beta }}_1} \cdot {\rm{log}}( {{\rm seq.bia}{{\rm s}_i}} )}}$, where ${\rm seq.bia}{{\rm s}_i}$ is the sequencing bias for inter-chromosomal pair $i$.

In the above model, we use chimeric data to estimate the noise level, i.e. random pairing, of inter-chromosomal pairs. The reason is that we can assume that the noise level of inter-chromosomal pairs is the same as that of chimeric pairs (see ChIA-PET workflow in (1)). In addition, we fit a positive Poisson regression to chimeric data, and this is based on our observation that ${\rm{log}}( \lambda )$ of chimeric pair data increases with ${\rm log}( {{\rm seq.bias}} )$ almost linearly in the two real datasets (See Supplementary Figure S2 in the Supplementary Data).

To estimate the positive Poisson parameter ${\lambda _k}$ for the intra-chromosomal pair $k$ ($1 \le k \le {n_{{\rm intra}}}$), we first create an auxiliary count table that consists of counts for two sets of pairs of anchor regions. The first set of pairs are those intra-chromosomal pairs with observed count being 1. The second set is of all pairs of two anchor regions that satisfy: (i) both anchor regions are on the same chromosome; (ii) both anchor regions appear in at least one intra-chromosomal pair; and (iii) the observed count for the pair is zero. Then, we fit a logistic regression

\begin{eqnarray*} \log \left( {\frac{{{p_l}}}{{1 - {p_l}}}} \right) &=& {\alpha _0}\ + {\alpha _1} \cdot \log \left( {{\rm seq.bia}{{\rm s}_l}} \right) \nonumber \\ &&+ {\alpha _2} \cdot {\rm{log}}\left( {{\rm distanc}{{\rm {\rm e}}_l}} \right) \end{eqnarray*}

to the auxiliary counts, where $l\ = \ 1,\ 2,\ \cdots ,\ {n_{{\rm auxiliary}}}$ is the index for the pairs in the auxiliary count table (${n_{{\rm auxiliary}}}$ is the number of pairs in the auxiliary count table), ${p_l}$ is the probability of observing count 1 for pair $l$, ${\rm seq.bia}{{\rm s}_l}$ is the sequencing bias of the pair $l$ and ${\rm distanc}{{\rm e}_l}$ is the genomic distance between the two anchor regions. Finally, we use the estimated values of parameters ${\hat{\alpha }_0}$, ${\hat{\alpha }_1}$ and ${\hat{\alpha }_2}$ to estimate the ${\lambda _k},$ i.e. ${\hat{\lambda }_k} = {e^{{{\hat{\alpha }}_0} + {{\hat{\alpha }}_1} \cdot \log ( {{\rm seq.bia}{{\rm s}_k}} ) + {{\hat{\alpha }}_2} \cdot \log ( {{\rm distanc}{{\rm {\rm e}}_k}} )}}$, where ${\rm seq.bia}{{\rm s}{\rm }_k}$ and ${\rm distanc}{{\rm e}_k}$ are the sequencing bias and the genomic distance between two anchor regions of the intra-chromosomal pair $k$.

The ${n_{{\rm auxiliary}}}$ pairs in the auxiliary count table serve as the noise in intra-chromosomal pairs. We assume that the pairs with count being 0 or 1 are very likely to be noise pairs (i.e. pairs of anchor regions with no interaction). Such an assumption is common to the existing tools, e.g. the ChIA-PET tool, which filter out potential pairs with count being 1 as noise by default. Given this assumption, each of the ${n_{{\rm auxiliary}}}$ pairs, under our null model, then follows a pair-specific Poisson distribution (not a positive Poisson distribution, as we allow zero counts here). For the auxiliary pair $l$, we have

\begin{eqnarray*} \log \left( {\frac{{{p_l}}}{{1 - {p_l}}}} \right) &=& \log \left( {\frac{{P\left( {{\rm count}\ {\rm of}\ {\rm pair}\ l\ = 1} \right)}}{{P\left( {{\rm count}\ {\rm of}\ {\rm pair}\ l = 0} \right)}}} \right) \nonumber \\ &=& \log \left( {\frac{{{\lambda _l}{e^{{\lambda _l}}}}}{{{e^{{\lambda _l}}}}}} \right)\ = \ {\rm{log}}\left( {{\lambda _l}} \right) \end{eqnarray*}

Because of that, we observed that ${\rm{log}}( {\frac{p}{{1 - p}}} )$ increases almost linearly with both of ${\rm log}( {{\rm seq.bias}} )$ and ${\rm log}( {{\rm distance}} )$ in both real datasets for testing (See Supplementary Figure S3 in the Supplementary Data), we fit a logistic regression to the auxiliary count data to estimate ${\lambda _k}$ using ${\rm seq.bia}{{\rm s}_k}$ and ${\rm distanc}{{\rm{\rm e}}_k}$. Note that we use the anchor regions that appear in at least one intra-chromosomal pair to construct the auxiliary table. This is because those pairs are more relevant to estimate the pair-specific positive Poisson parameter for intra-chromosomal pairs.

### Anchor regions and single-end alignment extension

To build anchor regions from peaks detected by MACS2 (i.e. to extend small peaks and to merge peaks next closely to each other) in step 5, we first estimate the minimum anchor length ${l_{{\rm ma}}}$ from input data (see Supplementary Data for details). Next, we extend all small peaks (i.e. peaks with length less than ${l_{{\rm ma}}}$) into regions with length equal to ${l_{{\rm ma}}}$ (the extension is done to both ends of a peak with an equal extension). Then, we merge any (possibly extended) peaks with gap (i.e. number of base pairs between the peaks) less than ${l_{{\rm ma}}}$. The resulted regions are anchor regions.

To extend single-end alignments to typical length of sequencing fragments in step 5, we first estimate the typical fragment length ${l_{{\rm fragment}}}$ (should be < ${l_{{\rm ma}}}$) from the data (see Supplementary Data for details). Then we extend single-end alignments to the length ${l_{{\rm fragment}}}$ in a 5′ to 3′ manner. Note that these single-end alignments are processed alignments, i.e. the orientations of these alignments have been reversed in step 3.

## RESULTS

We used two real datasets: the K562 RNA polymerase II data and MCF7 RNA polymerase II data in (3), to evaluate and compare ChIAPoP with the four existing methods: HG, MICC, ChiaSig and mango.

By default, all methods, except ChIAPoP, impose a cutoff on the count of potential pairs of anchor regions, so that only potential pairs with counts no less than the cutoff can be reported as significant pairs. The count cutoff is usually set to be 2 and 3 for small and large datasets, respectively. Because both our testing datasets are large (>75 million read pairs), we used the count cutoff 3 for all methods. Since ChIAPoP does not use the count cutoff by itself, the cutoff was imposed to the potential pairs after the *P*-values were obtained, and the FDR-adjustment (Benjamini–Hochberg Procedure) was then applied to the filtered pairs.

In all data analyses, we used the human genome hg19 as the reference genome to facilitate later evaluation with Hi-C and ChIP-Seq data, and the FDR cutoff 0.05 was used to call significant pairs. For more details of data analyses, please see the Supplementary Data.

### Goodness of fit for ChIAPoP

**Figure 1.**
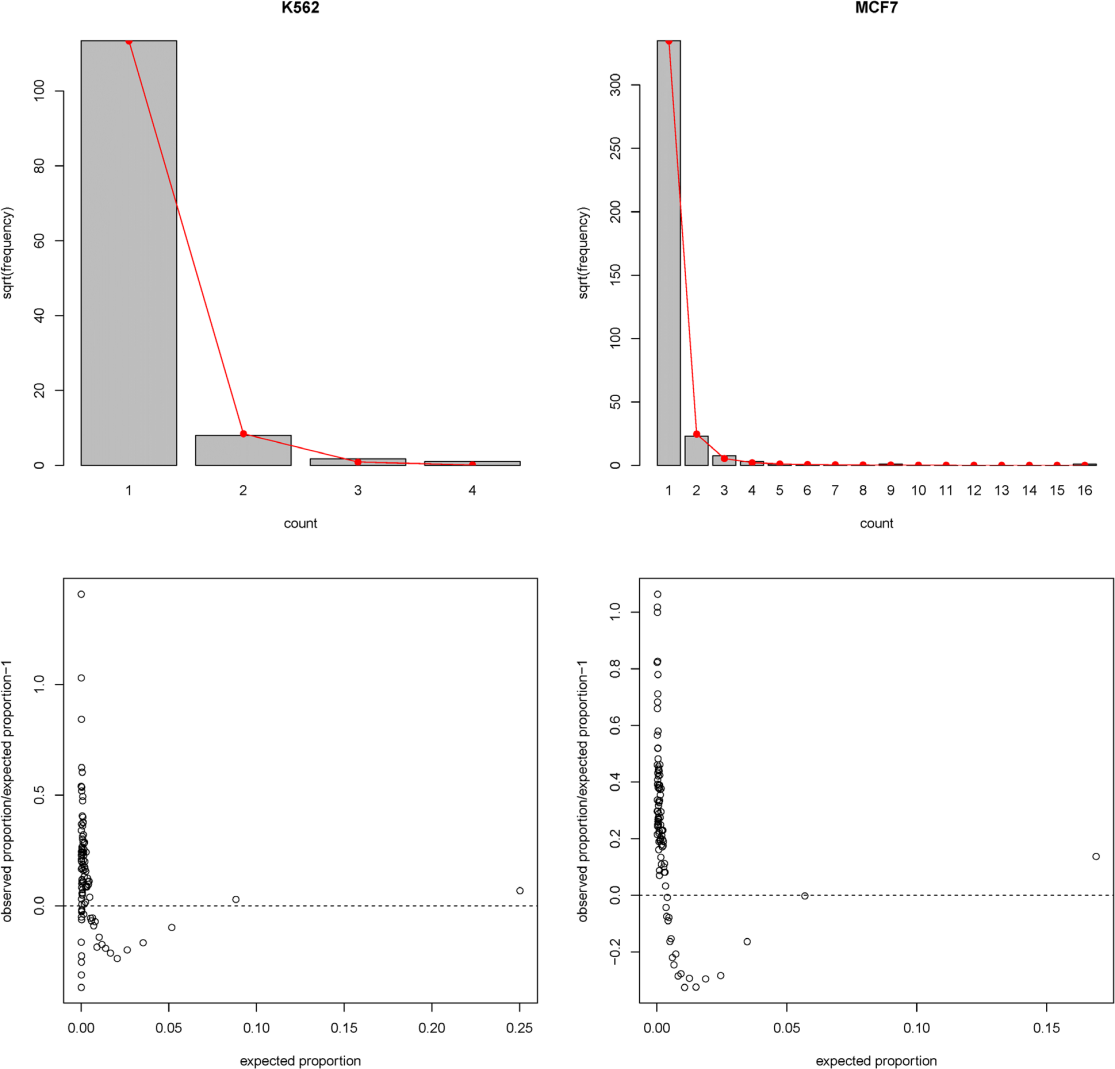
Goodness of fit for ChIAPoP in the K562 and MCF7 ChIA-PET datasets. Top: rootograms for the positive Poisson regressions that were applied on the chimeric count data for the two ChIA-PET datasets. A rootogram is a bar plot of observed frequencies overlaid with a curve of expected frequencies, both in square root scale. Bottom: scatter plots for the logistic regressions that were applied to the auxiliary count data for the two datasets. Each scatter plot shows the ratios of the observed proportions of 1s to the expected proportions of 1s, subtracted by 1 (*y*-axis), and the expected proportions of 1s (*x*-axis), for 100 bins of observations in the corresponding logistic regression. The 100 bins of observations were obtained by the 100-quantiles of their fitted probabilities of 1.

As shown in Figure 1, ChIAPoP achieved a good fit of the data for both datasets. The top two plots are the rootograms (19) for the positive Poisson regressions that were applied on the chimeric count data from the two datasets. A rootogram is a bar plot of observed frequencies that is overlaid with a curve of expected frequencies, both in square root scale. The two rootograms indicate that positive Poisson model fits both data well. The bottom two plots are the scatterplots for the logistic regressions of the auxiliary count data for the two datasets. Each scatterplot plots the ratios of observed and expected proportions of 1s, subtracted by 1, against the expected proportions of 1s, for the 100 bins of observations in the corresponding logistic regression. The 100 bins of observations were obtained by the 100-quantiles of their fitted probabilities of 1. For each bin, the expected proportion of 1s is calculated as the average fitted probability of count being 1 for all observations in the bin. Again, the two scatter plots indicate that logistic model fits both data well.

### Comparison of numbers of significant pairs detected by different methods

**Figure 2.**
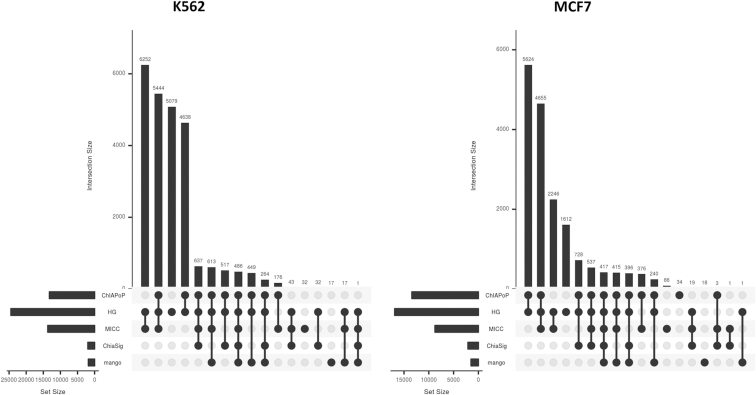
UpSet plots of significant pairs in the K562 and MCF7 ChIA-PET datasets. In each plot, the bottom left horizontal bars represent the numbers of significant pairs detected by different methods; and the vertical bars represent the sizes of different intersections of significant pair sets. The intersection that corresponds to a vertical bar is specified by the vertical black line with black filled circles under the bar. The UpSet plots are created by R package UpSetR.

Using the same FDR cutoff, ChIAPoP, on average, detected more significant pairs than MICC, ChiaSig and mango. ChIAPoP detected 13224 significant pairs in the K562 dataset and 13425 significant pairs in the MCF7 dataset, both of which are close to those detected by MICC (13701 and 8755), and are more than those detected by ChiaSig (1980 and 2101) and mango (1847 and 1487). HG found the highest numbers of significant pairs (24472 for K562 data and 16890 for MCF7 data). However, those significant pairs include almost all potential pairs with count $ \ge$ 3 (the proportions are 99.2 and 97.1% for K562 and MCF7, respectively). The number of significant pairs detected by both ChIAPoP and ChiaSig (or mango) is substantially higher than that detected by both MICC and ChiaSig (or mango) in both datasets, as shown in Table 1. Such a higher degree of consistency with the conservative ChiaSig and mango indirectly indicates that ChIAPoP is likely more accurate than MICC. To show intersections of sets of significant pairs detected by different methods and their sizes, we plotted the UpSet plots (20) for the two datasets in Figure 2. In each plot, the horizontal bars represent the sizes of the sets of significant pairs detected by different methods; and the vertical bars represent the sizes of different intersections of significant pair sets. For each vertical bar, the corresponding intersection is specified by the vertical black line with black filled circles under the bar.

**Table 1.** Comparison of numbers of significant pairs detected by two methods (one method is either ChIAPoP or MICC, and the other method is either ChiaSig or mango) in the K562 and MCF7 ChIA-PET datasets

	ChiaSig		mango	
	ChIAPoP	MICC	ChIAPoP	MICC
K562	1904 (0.962)	1167 (0.589)	1812 (0.981)	1117 (0.605)
MCF7	2081 (0.990)	977 (0.465)	1468 (0.987)	832 (0.560)

In a parenthesis is the proportion of the corresponding significant pairs in all significant pairs detected by the more conservative method (either ChiaSig or mango).

### Comparison of pair rankings by Aggregate Peak Analysis (APA)

It is hard if not impossible, without knowing the underlying truth, to get good estimates of detection sensitivity and specificity or other direct accuracy measures, so we evaluated the rankings of potential pairs by related validation data for comparison and assessment of different methods. Using related Hi-C data, we created Aggregate Peak Analysis (APA) plots and found that the ranking of potential pairs by ChIAPoP is better than those by other methods. We used FDR-adjusted *P*-values to rank the pairs for each method, except for ChiaSig which only outputs *P*-values. For HG and ChIAPoP, we also used *P*-values to break the ties among the rankings of adjusted *P*-values. Because these two methods use Benjamini–Hochberg procedure to adjust the *P*-values, the final rankings are equivalent to the rankings of *P*-values.

**Figure 3.**
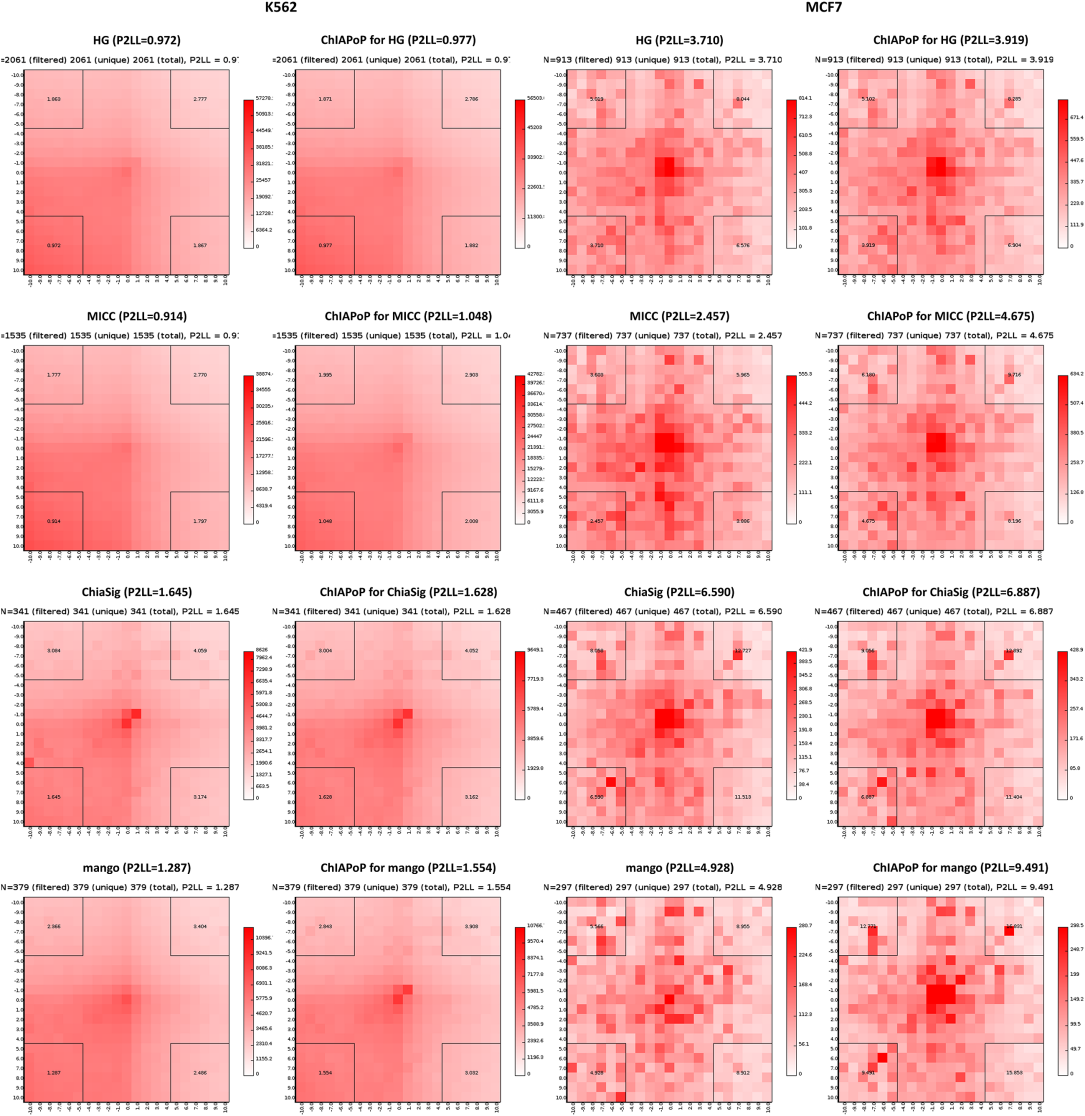
APA plots for the comparison of significant pairs detected by each existing method, and the corresponding ‘significant’ pairs detected by ChIAPoP. Each plot can be summarized by the APA score P2LL, the ratio of the central pixel to the mean of the mean of the pixels in the lower left corner. A higher P2LL indicates a better validation by the corresponding Hi-C data.

The APA plots were created by juicer tools (21) in 10 kb resolution, as shown in Figure 3. For each cell line, we created eight APA plots: four plots for existing methods, i.e. HG, MICC, ChiaSig and mango, and four corresponding comparison plots for ChIAPoP. Each such APA plot aggregates the Hi-C signal surrounding anchor regions ($ \pm 100$ kb) across all pairs in the corresponding set. The Hi-C signal used for the K562 cell line is from a high resolution Hi-C dataset in (22) and the Hi-C signal for the MCF7 cell line is from a Hi-C dataset in (23). For each cell line, as the juicer tools only uses intra-chromosomal pairs with distance greater than 300 kb to create APA plots by default, the set of pairs that was used to create the APA plot for an existing method is the set of significant intra-chromosomal pairs reported by the method with distance >300 kb; and the set of pairs that was used to create the APA plot for the corresponding comparison with ChIAPoP is the set of same (with respect to the method) number of intra-chromosomal pairs (filtered, i.e. with count $ \ge$ 3) with distance >300 kb, which were selected according to their ranks of ChIAPoP *P*-values, that is, the ones with the smallest *P*-values. As recommended in (22), we used the APA score P2LL, the ratio of the central pixel (a pixel represents a 10 kb $ \times$ 10 kb square) to the mean of the mean of the pixels in the lower left corner (a 6 pixel $ \times$ 6 pixel region), to summarize the APA plots. A higher P2LL indicates a better validation. Based on P2LL, we found that ChIAPoP pair ranking is better than those by other methods in both datasets, except for ChiaSig in K562 data, where P2LL for ChiaSig is 1.645 and P2LL for ChIAPoP is 1.628. However, we found that, in the APA plot for ChiaSig in K562 data, the left-most pixel right above the lower left corner has a strong aggregated Hi-C signal (which is comparable to the signal of the central pixel) compared to nearby pixels. This indicates a potential problem with the ChiaSig result, as one would usually expect a weak signal for this pixel.

We also compared the pair rankings of the five methods for the two ChIA-PET datasets using the cumulative APA plots (9), at the resolution 10 and 5 kb. The results are shown in the Supplementary Figure S4 in the Supplementary Data. In each cumulative APA plot, the five curves represent the five methods. Each curve demonstrates how the P2LL changes as the number of top pairs reported by the corresponding method increases. For resolution 10 kb (5 kb), the P2LL values were calculated in a cumulative way by adding 50 (100) distance-filtered pairs at a time, starting at the top 200 distance-filtered pairs. The P2LL values were calculated by the juicer tools using the default settings for each resolution. From the plots, we found that ChIAPoP is always one of the best performing methods for both datasets when evaluated at both resolutions, and that it is the only such method.

### Comparison of pair rankings by CTCF enrichment and CTCF motif orientation analyses

The DNA interactions are mostly related to CTCF binding and a pair of CTCF motifs involved in an intra-chromosomal DNA interaction is typically in a convergent orientation, that is, the two motifs are on different strands with the one with a smaller genomic coordinate on the reference strand (22). We performed CTCF enrichment analyses and CTCF motif orientation analyses to compare the rankings of potential pairs by ChIAPoP and the other methods. We found that ChIAPoP is as good as, if not better than, the other methods.

**Figure 4.**
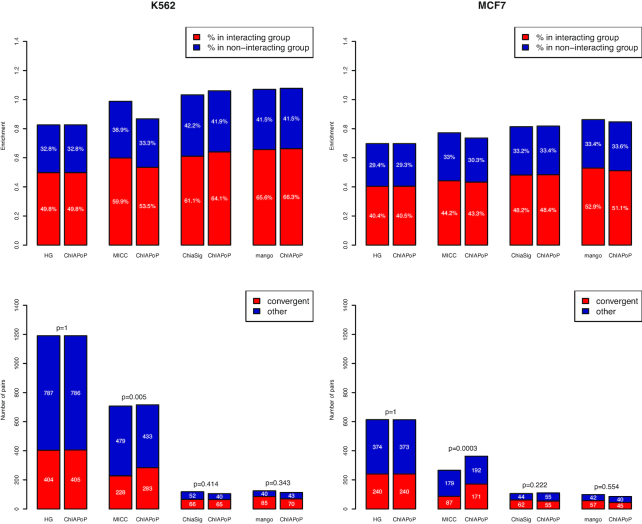
CTCF enrichment and CTCF motif orientation analyses in the K562 and MCF7 ChIA-PET datasets. Top: bar plots for CTCF enrichment analyses for the two ChIA-PET datasets. For each bar, the red part and the blue part represent the percentage of anchor regions that overlap with CTCF peaks in the interacting group and the percentage of anchor regions that overlap with CTCF peaks in the non-interacting group, respectively. Here the two groups were determined by the significant pairs reported by an existing method (HG, MICC, ChiaSig or mango), or by the corresponding set of ChIAPoP ‘significant’ pairs. Bottom: bar plots for CTCF motif orientation analyses for the two ChIA-PET datasets. For each bar, the red part and the blue part represent the number of significant intra-chromosomal pairs with two unique motifs in convergent orientation and the number of significant intra-chromosomal pairs with two unique motifs in other orientations, respectively. Here the significant pairs were reported by an existing method (HG, MICC, ChiaSig or mango), or were the corresponding ChIAPoP ‘significant’ pairs. The Fisher exact *P*-values shown in the figures are for the tests of proportions of motifs with convergent orientation between an existing method and ChIAPoP.

In the CTCF enrichment analyses, we investigated CTCF enrichment in anchor regions of the two groups: those that involve significant pairs and those that do not. If the significant pairs are more likely of true interactions than those not significant, the CTCF enrichment of anchor regions that involve significant pairs is expected to be higher. For the CTCF enrichment analyses, we used the CTCF-peak regions from the ENCODE ChIP-Seq datasets ENCFF681OMH and ENCFF559HEE for K562, and ENCFF720OXG and ENCFF990LUT for MCF7. For each cell line and an existing method, i.e. HG, MICC, ChiaSig or mango, we divided anchor regions into two groups: those that involve the significant pairs reported by the method (interacting group) and those that do not (non-interacting group). Then we calculated the percentage of anchor regions that overlap with the CTCF-peak regions in each of the two groups. To make a fair comparison between the method and ChIAPoP, we created a corresponding set of ChIAPoP ‘significant’ pairs by selecting the same (with respect to the method) number of potential pairs (filtered, i.e. with count ≥3) according to their ranks of ChIAPoP *P*-values, i.e. those with smallest *P*-values, and then repeated the above CTCF enrichment analysis with this set of ‘significant’ pairs. Notice that mango only reports intra-chromosomal pairs, so we only selected intra-chromosomal pairs to construct the corresponding set of ChIAPoP ‘significant’ pairs when we compared between mango and ChIAPoP. In total, we performed eight enrichment analyses for each cell line and the results are summarized as bar plots in Figure 4. From the figure, we found that ChIAPoP was better than or comparable to the other methods in both datasets in pair ranking. Here ‘better’ means a higher percentage of anchor regions that overlap with the CTCF-peak regions in the interacting group and lower percentage of anchor regions that overlap with the CTCF-peak regions in the non-interacting group. Also, we found the ChiaSig and mango results are better than MICC and HG results, as these two methods only reported the strongest signals.

In CTCF motif orientation analyses, we investigated the CTCF motif orientation for the significant intra-chromosomal pairs for different methods. If the detected significant intra-chromosomal pairs are true signals, we would expect to see the associated CTCF motifs in convergent orientation more often than in other orientations. For the analyses, we determined the CTCF motifs in each cell line as the following. First, we obtained the predicted CTCF motifs from PWMScan (24), a web server (https://ccg.vital-it.ch/pwmscan/) for scanning of a reference genome for high-scoring matches to a given position weight matrix (PWM). In the scan, we used the hg19 as the reference genome and a PWM that is derived from the CTCF-binding profile (ID: MA0139.1) in the database JASPAR CORE 2018 vertebrates (25). The parameters used in the PWMScan are the default values. Second, for each cell line, we filtered the predicted CTCF motifs using the CTCF-peak regions from the corresponding ENCODE ChIP-Seq data (the same datasets that we used in the CTCF enrichment analyses). That is, we only kept those that overlap with the CTCF-peak regions. These predicted CTCF motifs were used for the motif orientation analyses for the corresponding cell line. After obtaining the CTCF motifs in each cell line, for an existing method we then counted the number of significant intra-chromosomal pairs with each of two anchor regions overlaps with a unique CTCF motif and the number of such significant pairs with the corresponding CTCF motif orientation being convergent. To make a fair comparison between the method and ChIAPoP, we again created a corresponding set of ChIAPoP ‘significant’ intra-chromosomal pairs by selecting the same (w.r.t the method) number of intra-chromosomal pairs (filtered, i.e. with count $ \ge$ 3) according to their ranks of ChIAPoP *P*-values, i.e. those with smallest *P*-values, and then repeated the above CTCF motif orientation analysis with this set of ‘significant’ intra-chromosomal pairs. In total, we performed eight CTCF motif orientation analysis for each cell line and the results are summarized as bar plots in Figure 4. Again, we found that ChIAPoP pair ranking is as good as the ranking by other methods, if not better, in both datasets. Fisher exact tests (shown in the figure) on the proportions show that ChIAPoP rankings are better than MICC rankings, and are not significantly different from other rankings.

## DISCUSSION

ChIA-PET is a widely used assay method to study genome-wide chromatin interactions mediated by a protein of interest. Here we proposed a new approach and developed a new analysis pipeline, ChIAPoP, to identify real chromatin interactions from ChIA-PET data of the original protocol. It is a complete analysis pipeline that includes linker removal, read alignment, alignment processing, anchor region detection and DNA interaction detection. Using two real ChIA-PET datasets, we demonstrated that our new models were effective in fitting data, and ChIAPoP performed better than or at least comparable to the top existing methods in identifying real chromatin interactions.

ChIAPoP is able to take the full advantage of ChIA-PET chimeric data. Although some of the existing tools also use chimeric data, they do no fully use the information in the data. For example, the ChIA-PET tool uses chimeric data only for determining the count cutoff for data filtering. In contrast, ChIAPoP directly fits the chimeric data with its positive Poisson model for estimating noise level for inter-chromosomal pairs, and fits with the logistic model for intra-chromosomal pairs.

Mango is a relatively conservative method, which is likely to be the main reason that it detected fewer significant pairs than other methods in our comparisons. Mango considers only intra-chromosomal pairs, and it may be also part of the reason. Among all significant pairs, the portion of significant inter-chromosomal pairs detected by the other methods, however, is small in both datasets (mostly ranging between 1.4 and 7.2% except ChiaSig with 12.1% in K562). So, even if mango were able to detect inter-chromosomal pairs, the comparison results would not change much. In all comparisons, ChiaSig tool was applied to the two groups of potential pairs (inter-chromosomal and intra-chromosomal) separately in order to be consistent with (8). We also applied ChiaSig tool to all potential pairs, however, we only got a few hundred significant pairs in each dataset.

For both datasets, the comparisons between ChIAPoP and HG by APA and CTCF analyses did not fully reflect the advantage of ChIAPoP over HG, as HG was used as the reference method and almost the full data (all potential pairs with count ≥3) were selected by HG including many non-significant ones in ChIAPoP. Therefore, we performed similar comparisons between the two methods, but used ChIAPoP as the reference method. The results show that the pair ranking of ChIAPoP is better than that of HG. Please see Supplementary Figures S5 and S6 in the Supplementary Data for more details.

The current version of ChIAPoP main analysis pipeline only supports reads data from the original ChIA-PET protocol, which is still widely used. Nevertheless, we do include a separate function in our ChIAPoP R package to support data from the new ChIA-PET protocol. This function requires a count table for potential pairs and a table of sequencing bias of anchor regions as the input. For intra-chromosomal pairs, this function is identical to the step 6 in the ChIAPoP pipeline (estimating the pair specific positive Poisson parameters by a logistic regression). For inter-chromosomal pairs, this function estimates pair-specific positive Poisson parameters by another logistic regression (with a single independent variable $log( {seq.bias} )$), instead of a positive Poisson regression because there is no chimeric data for the improved protocol. The auxiliary count table is created in the similar way as creating the auxiliary count table for testing intra-chromosomal pairs. The two input tables for the function can be easily generated by using the output from other tools, e.g. ChIA-PET2.

## Supplementary Material

Supplementary Data
