# Supplementary material for: ChIAPoP: a new tool for ChIA-PET data analysis
Source: Nucleic Acids Res. 2019 Feb 8;47(7):e37. doi: 10.1093/nar/gkz062 (PMC6468250; doi:10.1093/nar/gkz062)

Title: ChIAPoP: A new tool for ChIA-PET data analysis

Weichun Huang, Mario Medvedovic, Jingwen Zhang and Liang Niu

SUPPLEMENTARY MATERIALS

I. **Supplementary Methods**
II. **Supplementary Figures**

I. Supplementary Methods

Estimation of minimum anchor length (l_{ma}) and typical fragment length ($l_{fragment}$)

To estimate l_{ma} and $l_{fragment}$, we need to consider the PETs that are from self-loops (i.e., the loops formed by ligating the two ends of single DNA fragments). By considering distances between the two tags (i.e., the distance between the midpoints of the two tags) in such PETs, we can estimate the distribution of DNA fragment length. Although we do not have the prior information about that whether a PET is from a self-loop or not, we observe that, if such a PET is from a self-loop, then the two tags must be aligned to two different strands of the same chromosome. Furthermore, if the reference genome is accurate (i.e., no insertions and deletions), the alignment of a PET that is from a self-loop can only be of type RF (reverse-forward, i.e., two tags are aligned to two different strands of the reference genome; and the one with the smaller start position is aligned to the reverse strand), while the alignment of a PET (with two tags aligned to the same chromosome) from non-self-loops can be of any type (i.e., FF, RR, FR or RF) with equal possibilities.

Because of the above observations, ChIAPoP proceeds as follows to estimate l_{ma} and $l_{fragment}$. In step 3, for each filtered intra-chromosomal regular alignment pair (before reversing the orientation of each single-end alignment), we record the type of the alignment pair (i.e., FF, RR, FR or RF) and the genomic distance between the two alignments (tags). Then, in step 5, for each bin of size 10 base pairs (e.g., $[0, 10)$, $[10, 20)$, etc.), we count the number of recorded alignment pairs with genomic distances that fall into the bin for each type. In this way, we get four numbers (n_{FF}, n_{RR}, n_{FR} and n_{RF}) for each bin. Next, we calculate the $(n_{FR} + n_{RF}) - (n_{FF} + n_{RR})$, which is the difference between the number of recorded alignment pairs with reads from different strands and the number of recorded alignment pairs with reads from the same strand (i.e., the estimated number of self-loops), for each bin and choose the bin with the value being maximized. Then $l_{fragment}$ is estimated as the sum of the right end point of the bin and a typical tag length (20 base pairs). To estimate l_{ma}, we consider the empirical cumulative density distribution (cdf) of genomic distances for the recorded pairs with type FR or RF (F_{diff}) and that for the recorded pairs with type FF or RR (F_{same}). We then choose l such that, empirically, self-loops with two tags of genomic distance $\leq l$ constitute 95% of all self-loops, i.e.,

$$\frac{F_{diff}(l) \cdot (N_{FR} + N_{RF}) - F_{same}(l) \cdot (N_{FF} + N_{RR})}{(N_{FR} + N_{RF}) - (N_{FF} + N_{RR})} = 95\%$$

, where $N_{FF}/N_{RR}/N_{FR}/N_{RF}$ is the total number of recorded alignment pairs with strand type FF/RR/FR/RF. Then l_{ma} is estimated as the sum of l and a typical tag length (20 base pairs). In the above process, users can also use n_{RF}, N_{RF}, $\frac{n_{FF}+n_{RR}}{2}$, $\frac{N_{FF}+N_{RR}}{2}$ and F_{RF}, in the places of $n_{FR} + n_{RF}$, $N_{FR} + N_{RF}$, $n_{FF} + n_{RR}$, $N_{FF} + N_{RR}$ and F_{diff}, respectively, where F_{RF} is the cdf of genomic distances for the recorded pairs with type RF. The 95% cutoff that is used to determine l_{ma} can also be adjusted by users.

Details of data analyses for K562 and MCF7 RNA polymerase II data

The raw read data was downloaded from European Nucleotide Archive with run accession numbers SRR372747, SRR372748 (two K562 replicates), SRR372741 and SRR372742 (two MCF7 replicates). The two replicates for each cell line are combined into a single experiment. The ChIAPoP result was obtained by the ChIAPoP pipeline. The HG result was obtained using the regular count table created by the ChIAPoP pipeline. The MICC result was obtained by the MICC2.R function in ChIA-PET2 pipeline, using the regular count table created by the ChIAPoP pipeline. The ChiaSig result was obtained by the

ChiaSig software, using the regular count table created by the ChIAPoP pipeline. In order to be consistent with the original ChiaSig publication, the ChiaSig software was run separately on the two groups of regular pairs, i.e., inter-chromosomal and intra-chromosomal pairs. The mango result was obtained by the mango software, using a file with all anchor regions created by the ChIAPoP pipeline as the …slopPeak file.

The commands used to download data and to perform ChIAPoP analyses are shown as below:

K562

```
mkdir K562
cd K562

wget ftp://ftp.sra.ebi.ac.uk/vol1/fastq/SRR372/SRR372748/SRR372748_1.fastq.gz
wget ftp://ftp.sra.ebi.ac.uk/vol1/fastq/SRR372/SRR372748/SRR372748_2.fastq.gz
wget ftp://ftp.sra.ebi.ac.uk/vol1/fastq/SRR372/SRR372747/SRR372747_1.fastq.gz
wget ftp://ftp.sra.ebi.ac.uk/vol1/fastq/SRR372/SRR372747/SRR372747_2.fastq.gz

gunzip SRR372748_1.fastq.gz
gunzip SRR372748_2.fastq.gz
gunzip SRR372747_1.fastq.gz
gunzip SRR372747_2.fastq.gz

cat SRR372747_1.fastq SRR372748_1.fastq > K562_1.fastq
cat SRR372747_2.fastq SRR372748_2.fastq > K562_2.fastq

(in R)
library(ChIAPoP)
result<-pop.pipeline(“K562_1. fastq”, “K562_2. fastq”, “path/to/bowtie/hg19 index”)
```

MCF7

```
mkdir MCF7
cd MCF7

wget ftp://ftp.sra.ebi.ac.uk/vol1/fastq/SRR372/SRR372741/SRR372741_1.fastq.gz
wget ftp://ftp.sra.ebi.ac.uk/vol1/fastq/SRR372/SRR372741/SRR372741_2.fastq.gz
wget ftp://ftp.sra.ebi.ac.uk/vol1/fastq/SRR372/SRR372742/SRR372742_1.fastq.gz
wget ftp://ftp.sra.ebi.ac.uk/vol1/fastq/SRR372/SRR372742/SRR372742_2.fastq.gz

gunzip SRR372741_1.fastq.gz
gunzip SRR372741_2.fastq.gz
gunzip SRR372742_1.fastq.gz
gunzip SRR372742_2.fastq.gz

cat SRR372741_1.fastq SRR372742_1.fastq > MCF7_1.fastq
cat SRR372741_2.fastq SRR372742_2.fastq > MCF7_2.fastq

(in R)
library(ChIAPoP)
result<-pop.pipeline(“MCF7_1. fastq”, “MCF7_2. fastq”, “path/to/bowtie/hg19 index”)
```

II. Supplementary Figures

Figure S1: The flow chart of ChIAPoP pipeline.

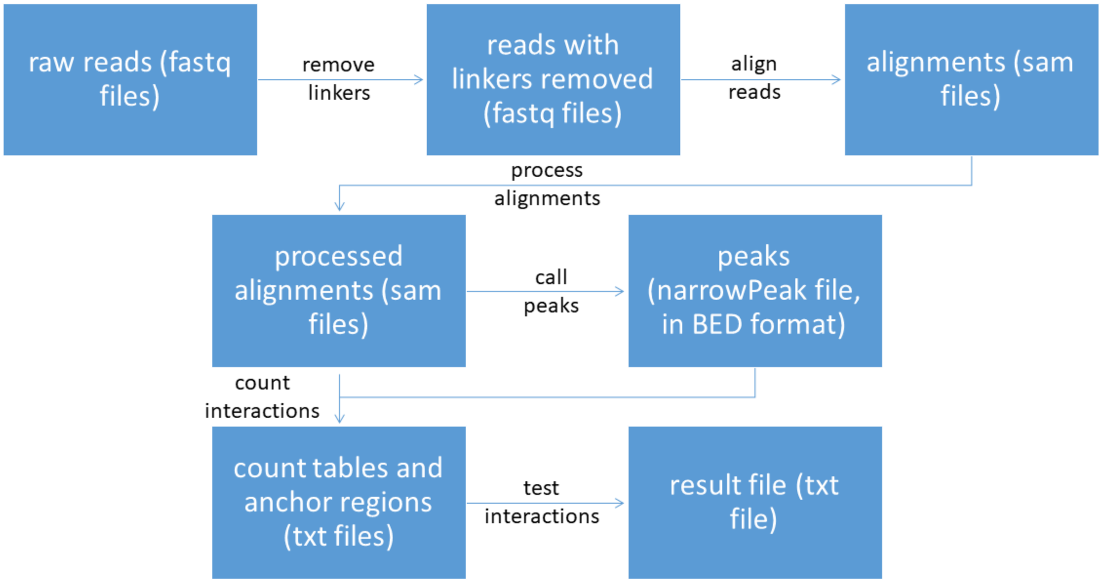

Figure S2: The Chimeric count data follows a roughly positive Poisson model in the two ChIA-PET datasets (K562 and MCF7). In each figure, we plot the estimated $\log \lambda$ at ten $log(seq.bias)$ values. The estimated $\log \lambda$ values and the ten $log(seq.bias)$ values were obtained as follows. First, we divided the chimeric pairs into 10 bins using the 10-quantiles of their $log(seq.bias)$ values. Then, we found the maximum likelihood estimator of the positive Poisson parameter (assuming that the parameter is the same for all observations in the same bin) in each bin. The $\log \lambda$ values were then the logarithms of the estimated λs, and the ten $log(seq.bias)$ values are the midpoint of each bin (the left end point of the first bin is defined as the minimum value of $log(seq.bias)$ values; the right end point of the last bin is defined as the maximum value of $log(seq.bias)$ values).

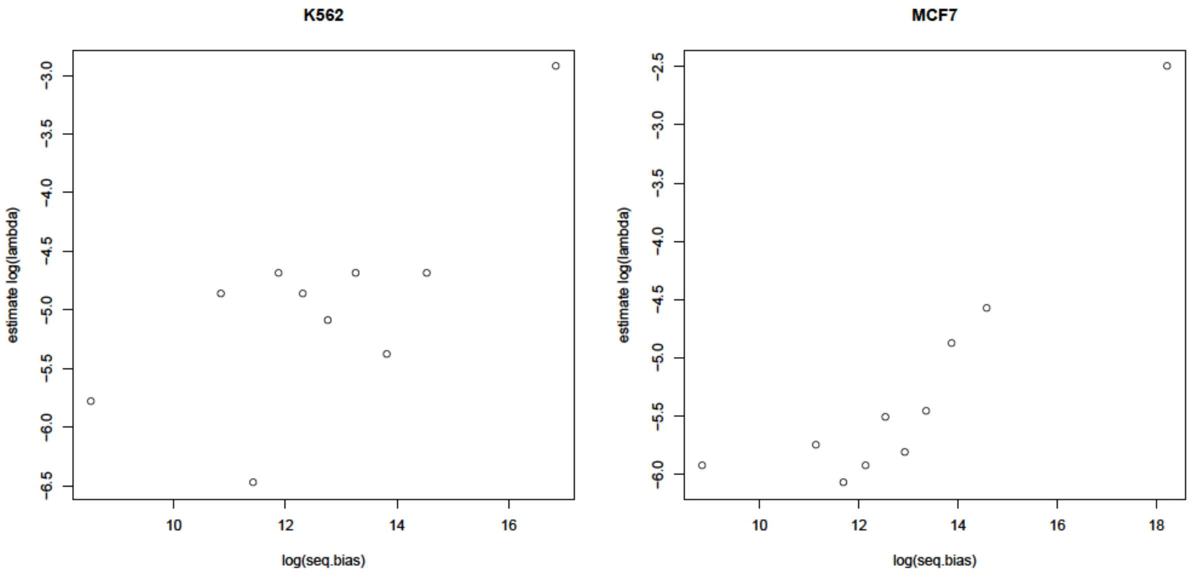

Figure S3: The auxiliary count data (binary data) follows roughly a logistic model in the two ChIA-PET datasets (K562 and MCF7). In the top two figures, we plotted the estimated $\log(p/(1-p))$ at 1000 $log(seq.bias)$ values for the two datasets. In the bottom two figures, we plotted the estimated $\log(p/(1-p))$ at 1000 $log(distance)$ values for the two datasets. The estimated $\log(p/(1-p))$ values and the 1000 $log(seq.bias)$ values for each of the two top figures were obtained as follows. First, we divided the auxiliary pairs into 1000 bins using the 1000-quantiles of their $log(seq.bias)$ values. Then, we found the empirical $\log(p/(1-p))$ values in each bin (i.e., let p = observed proportions of 1s in each bin). These empirical $\log(p/(1-p))$ values were then the estimated $\log(p/(1-p))$ values, and the 1000 $log(seq.bias)$ values are the midpoint of each bin (the left end point of the first bin is defined as the minimum value of $log(seq.bias)$ values; the right end point of the last bin is defined as the maximum value of $log(seq.bias)$ values). The estimated $\log(p/(1-p))$ values and the 1000 $log(distance)$ values for each of the two bottom figures were obtained similarly.

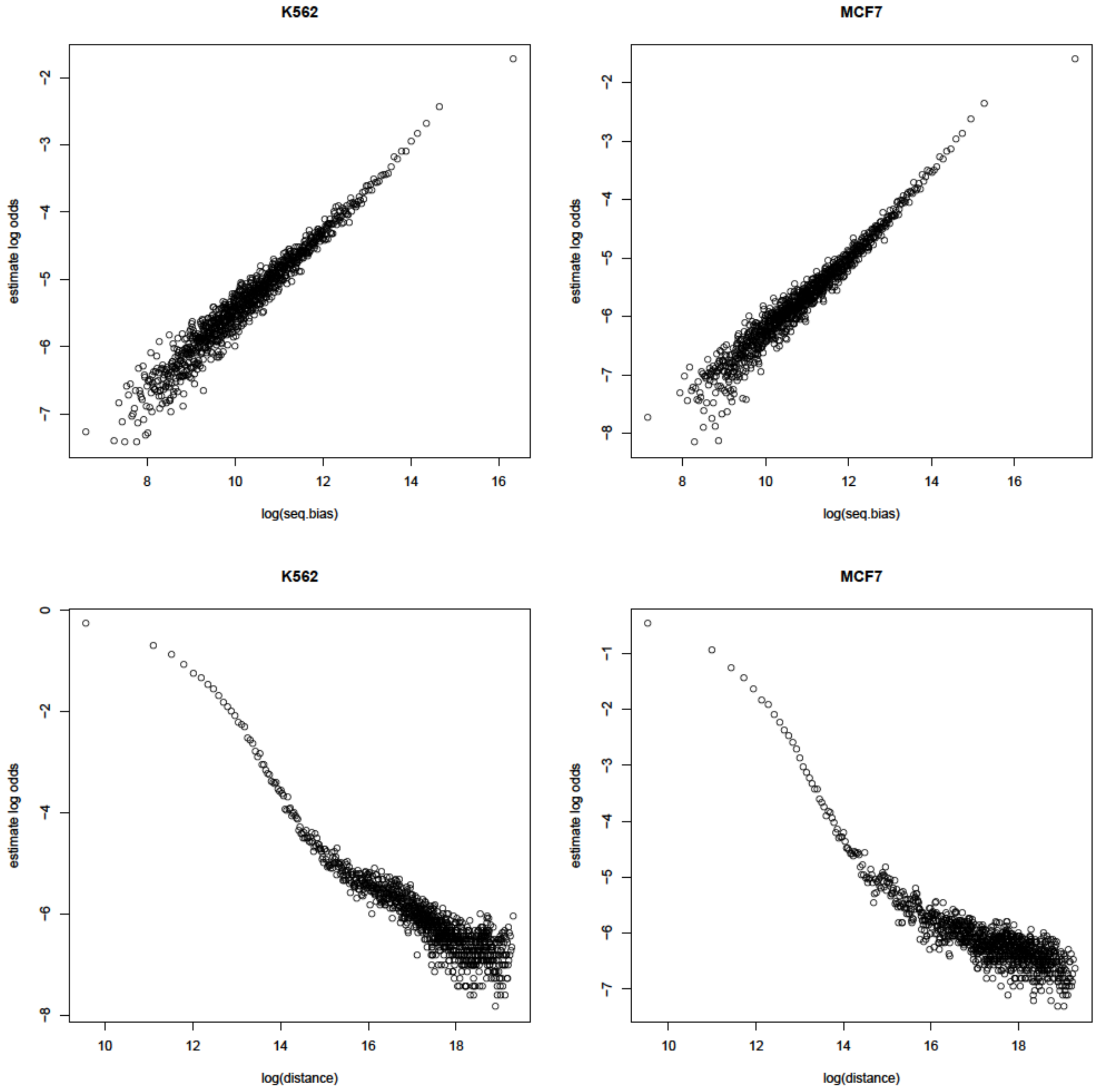

Figure S4: Cumulative APA plots for the comparison of the five methods. A curve in each plot demonstrates how the P2LL changes with the increase of the number of top pairs reported by the corresponding method. The dot on each curve shows the P2LL value for the set of distance-filtered significant pairs reported by the corresponding method. For resolution 10 kb (5 kb), the P2LL values were calculated in a cumulative way by adding 50 (100) distance-filtered pairs at a time, starting at the top 200 distance-filtered pairs. The P2LL values were calculated by the juicer tools using the default settings for each resolution.

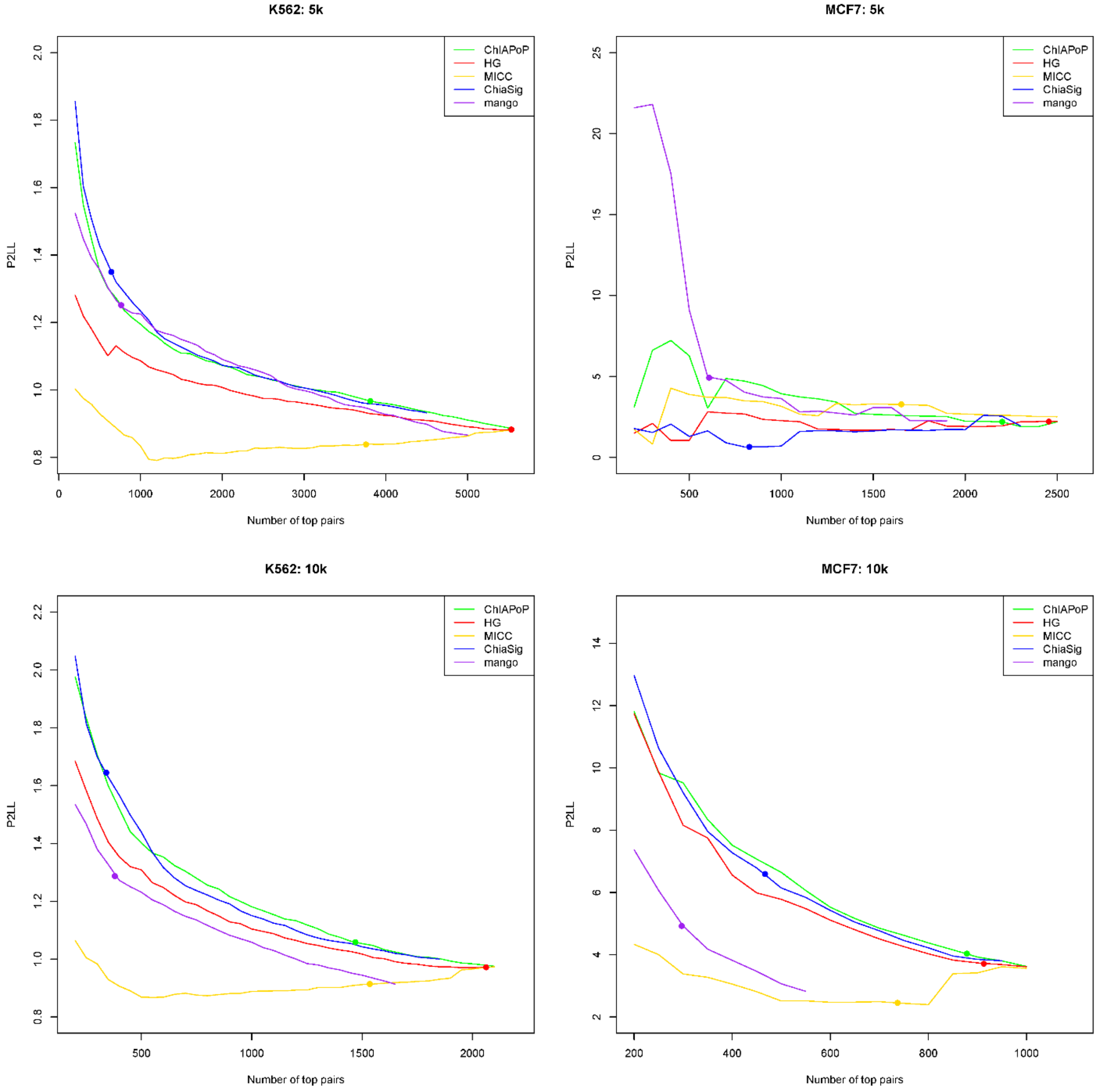

Figure S5: APA plots for the comparison of significant pairs detected by ChIAPoP, and the corresponding “significant” pairs detected by HG. Each plot can be summarized by the APA score P2LL, the ratio of the central pixel to the mean of the mean of the pixels in the lower left corner. A higher P2LL indicates a better validation by the corresponding Hi-C data.

K562

ChIAPoP (P2LL=1.059)

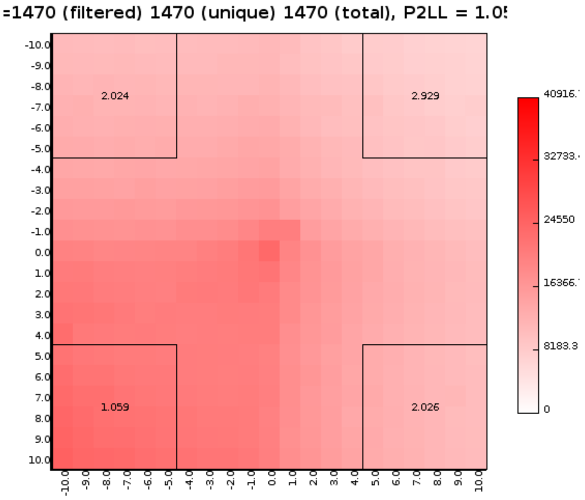

MCF7

ChIAPoP (P2LL=4.033)

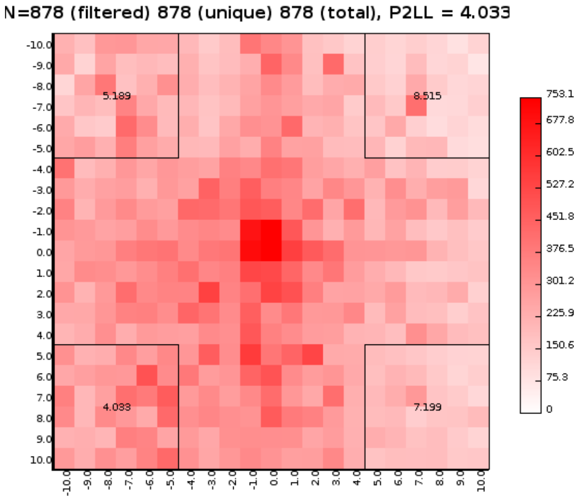

HG for ChIAPoP (P2LL=1.022)

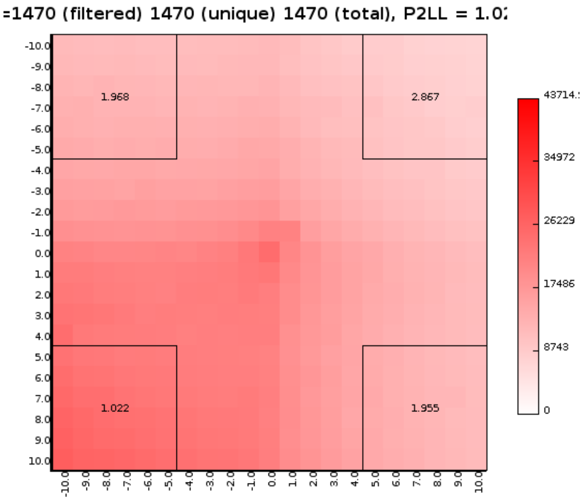

HG for ChIAPoP (P2LL=3.786)

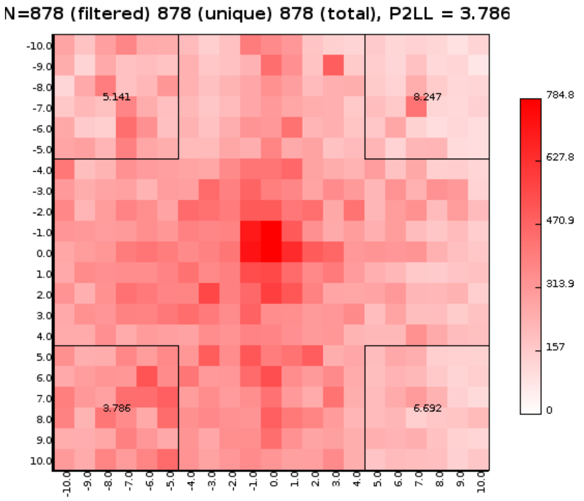

Figure S6: CTCF enrichment and CTCF motif orientation analyses in the K562 and MCF7 ChIA-PET datasets, for the comparison of ChIAPoP and HG. Top: bar plots for CTCF enrichment analyses for the two ChIA-PET datasets. For each bar, the red part and the blue part represent the percentage of anchor regions that overlap with CTCF peaks in the interacting group and the percentage of anchor regions that overlap with CTCF peaks in the non-interacting group, respectively. Here the two groups were determined by the significant pairs reported by ChIAPoP, or by the corresponding set of HG "significant" pairs. Bottom: bar plots for CTCF motif orientation analyses for the two ChIA-PET datasets. For each bar, the red part and the blue part represent the number of significant intra-chromosomal pairs with two unique motifs in convergent orientation and the number of significant intra-chromosomal pairs with two unique motifs in other orientations, respectively. Here the significant pairs were reported by ChIAPoP, or were the corresponding HG "significant" pairs. The Fisher exact p-values shown in the figures are for the tests of proportions of motifs with convergent orientation between ChIAPoP and HG.

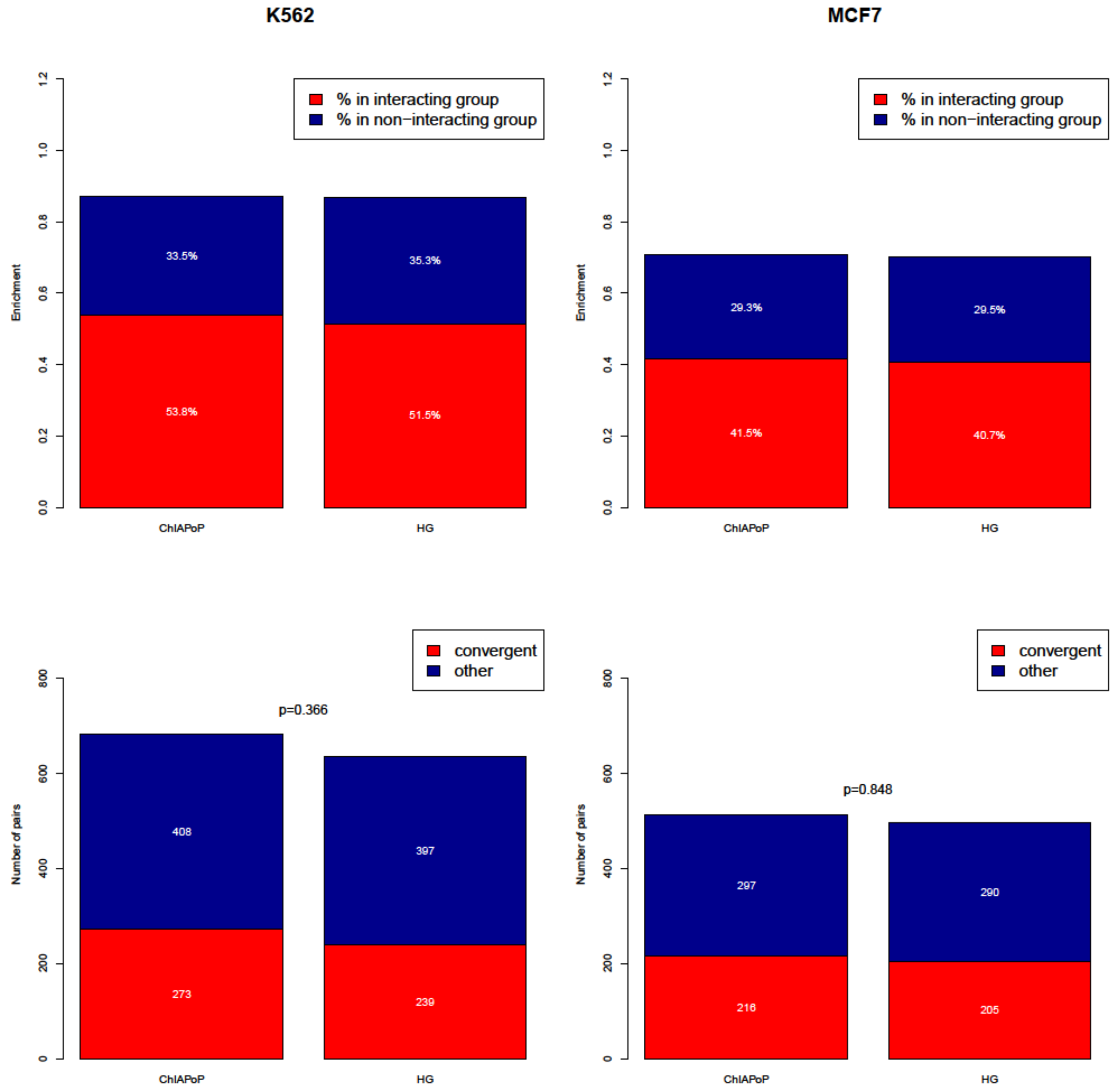

Supplement: Supplementary Data [file gkz062_supplemental_file.pdf]
